# A different lens on diagnosis: value of the CFI in asylum seekers’ psychiatric diagnostic assessment

**DOI:** 10.1192/bjo.2025.10828

**Published:** 2025-09-08

**Authors:** Lukas Claus, Bernard Sabbe, Sofie Bäärnhielm, Marianne Destoop, Meryam Schouler-Ocak, Mario Braakman, Seline van den Ameele

**Affiliations:** Collaborative Antwerp Psychiatric Research Institute (CAPRI), Universiteit Antwerpen (UA), Antwerp, Belgium; Department of Psychiatry, Vrije Universiteit Brussel (VUB), Universitair Ziekenhuis Brussel (UZ Brussel), Brussels, Belgium; Department of Clinical Neuroscience, Center for Psychiatry Research, Karolinska Institutet (KI), Stockholm, Sweden; Stockholm Health Care Services, Stockholm, Sweden; Transcultural Centre, Region Stockholm, Stockholm, Sweden; Multiversum Psychiatric Hospital, Boechout, Belgium; Department of Psychiatry and Psychotherapy, Psychiatric University Clinic of Charité at St. Hedwig Hospital, Berlin, Germany; Department of Criminal Law, Tilburg Law School, Tilburg University, Tilburg, The Netherlands; Department of Psychiatry and Medical Psychology, Brugmann University Hospital, Brussels, Belgium

**Keywords:** Asylum seekers, Cultural Formulation Interview, mental health, diagnosis, mixed methods

## Abstract

**Background:**

Asylum seekers face significant mental health challenges but underutilise mental health services and are at increased risk of misdiagnosis. The Cultural Formulation Interview (CFI) could be helpful by introducing individuals’ culture and context to psychiatric evaluation. However, its impact on the diagnostic process for asylum seekers remains underexplored.

**Aims:**

This study aims to evaluate the added value of the CFI in the psychiatric diagnostic assessment of asylum seekers.

**Method:**

A mixed-methods design was applied. Diagnostic shifts from the CFI were quantitatively described in 63 participating asylum seekers. The CFI’s value was explored using qualitative content analysis.

**Results:**

In about a third of cases, diagnoses were either confirmed (34.9%), changed (25.4%) or narrowed (33.3%), with notable shifts from depressive and psychotic disorders to either trauma- and stressor-related disorders or no psychopathology. Qualitative analysis revealed that the CFI enhanced understanding of participants’ experiences, including the impact of trauma, migration and social context. It provided insights into their strengths and therapeutic needs. The shift towards stress-related diagnoses and away from other common DSM categories reflects the CFI’s ability to provide a more nuanced, contextual understanding of asylum seekers’ mental health.

**Conclusion:**

This study underscored the CFI as a valuable tool in asylum seekers’ diagnostic assessment. The CFI facilitated a shift towards a more holistic, recovery-oriented approach. It prompted conceptual reflections on psychopathology in asylum seekers. The CFI presents a promising yet underutilised tool for addressing diagnostic challenges in cross-cultural settings. The findings highlight its potential for broader clinical implementation.

By the end of 2023, 110 million people had been forcibly displaced globally, including 6.9 million asylum seekers.^1^ Asylum seekers face numerous risk factors for mental health illness, including trauma, lack of shelter, uncertainty and the long duration of the asylum procedure.^2^ Although mental health illness is highly prevalent among asylum seekers,^3^ their use of mental health services is low compared with their need.^4,5^ This underutilisation may be explained by various barriers, such as lack of knowledge of the healthcare system, language barriers, distrust of authority, structural difficulties (financial limitations, precarity, lack of capacity …), social exclusion and differing beliefs and expectations about mental health and healthcare.^6,7^ In multicultural encounters, clinicians may struggle to interpret individuals’ diverse idioms of distress due to cultural differences in understanding and expressing psychological distress.^8^ Additionally, standard DSM diagnoses, based on decontextualised criteria, may fail to represent an individual’s experience (category fallacy).^9^ Different studies indicate that those from ethnic minorities, in particular refugees and recently arrived migrants, are at higher risk of misdiagnosis.^8,10,11^ When cultural factors are not adequately considered, incorrect diagnoses or misjudgements of illness severity can occur.^10^ This may cause communication barriers, hinder client engagement and prolong suffering, potentially leading to premature treatment termination.^12^ Greater attention to cultural and contextual factors may enhance diagnostic accuracy and treatment planning.^13^

The Cultural Formulation Interview (CFI) is a valuable tool through which to incorporate the individual’s perspective and social context into psychiatric evaluations, facilitating a narrative description of illness experiences.^14,15^ Previous studies evaluated the CFI among diverse migrant populations, excepting asylum seekers, and found it to be a feasible, acceptable and useful clinical tool.^12,15^ Lindberg et al emphasise the CFI’s importance in vulnerable and asymmetrical encounters with migrants or other marginalised groups.^16^ While the CFI’s implementation has been evaluated several times, research on its effect on clinical outcomes remains scarce.^12^ Two studies report improved patient–clinician communication due to CFI.^17,18^ Case reports suggest that CFI can alter clinical service delivery, diagnostic formulation and treatment adherence.^19–23^ Revisiting diagnostic categorisation using DSM-IV-TR’s Cultural Formulation method led to diagnostic changes in about half of the cases.^8,10^ Only one study has evaluated the impact of DSM-5 CFI on the diagnostic process, describing how use of the CFI might help identify depressive disorders in non-native-speaking subjects with a migration background.^24^

Despite the CFI’s goal of improving diagnostic accuracy, further research is still needed to determine whether it effectively achieves this.^12^ Although asylum seekers have specific mental health needs and face clear barriers that the CFI could help address, all existing studies on the CFI have excluded them. Therefore, this study aimed to evaluate the value of the CFI in the psychiatric diagnostic assessment of asylum seekers with mental health illnesses. Quantitatively, this study hypothesises that the CFI leads to diagnostic shifts in a significant proportion of asylum seekers, with a higher proportion of stress-related disorders post-CFI. Qualitatively, this study holds the working hypothesis that the CFI strengthens clinicians’ understanding of the nature, causes and remedies of asylum seekers’ mental suffering.

Box 1 Mental healthcare for asylum seekers in Belgium

In Belgium, asylum seekers are entitled to social, medical and psychological support. They have access to primary care, specialised services (including general psychological and psychiatric follow-up) as well as targeted support for specific situations such as torture, organised violence or gender-related issues. Several categorical care initiatives exist specifically for asylum seekers, including residential psychological support programmes, specialised psychiatric follow-up and day treatment for minors with severe psychological difficulties.^25^ However, various barriers impede access to mental healthcare.^6^

## Method

This study is part of a larger project focused on use of the CFI with asylum seekers, and applies a mixed-methods approach as outlined in a prepublished protocol paper.^26^

### Participants

This project was set in routine clinical care for asylum seekers in either a reception centre, hospital or therapeutic service. Participants were referred by a caregiver (healthcare or social worker) based on suspected mental illness. Inclusion criteria involved the need for a psychiatric referral, being in an ongoing asylum procedure in Belgium, the ability of oral communication and of giving written informed consent. Participants with severe cognitive disability, acute suicidality or intoxicated with, or in withdrawal of, substances were excluded. This study focused on young asylum seekers (15–29 years). A power analysis based on a supposed effect size of 0.25, with 10% precision at the 95% confidence level, indicated a minimum of 73 participants. From a qualitative perspective, a sample of 60 participants was considered sufficient to ensure adequate diversity. Accordingly, the target sample size was set at between 60 and 80 participants over 18 months. Due to time and funding constraints, recruitment was concluded following enrollment of 67 participants.^26^ All participants gave written informed consent, with the support of an interpreter if indicated. Because all minor participants were unaccompanied, they gave written consent themselves; 11 of 22 minor participants had a legal representative, whose agreement was also requested. The complete demographic characteristics of the participants are reported in Supplementary Table 1 available at https://doi.org/10.1192/bjo.2025.10828.

### Procedure

**Fig. 1:**
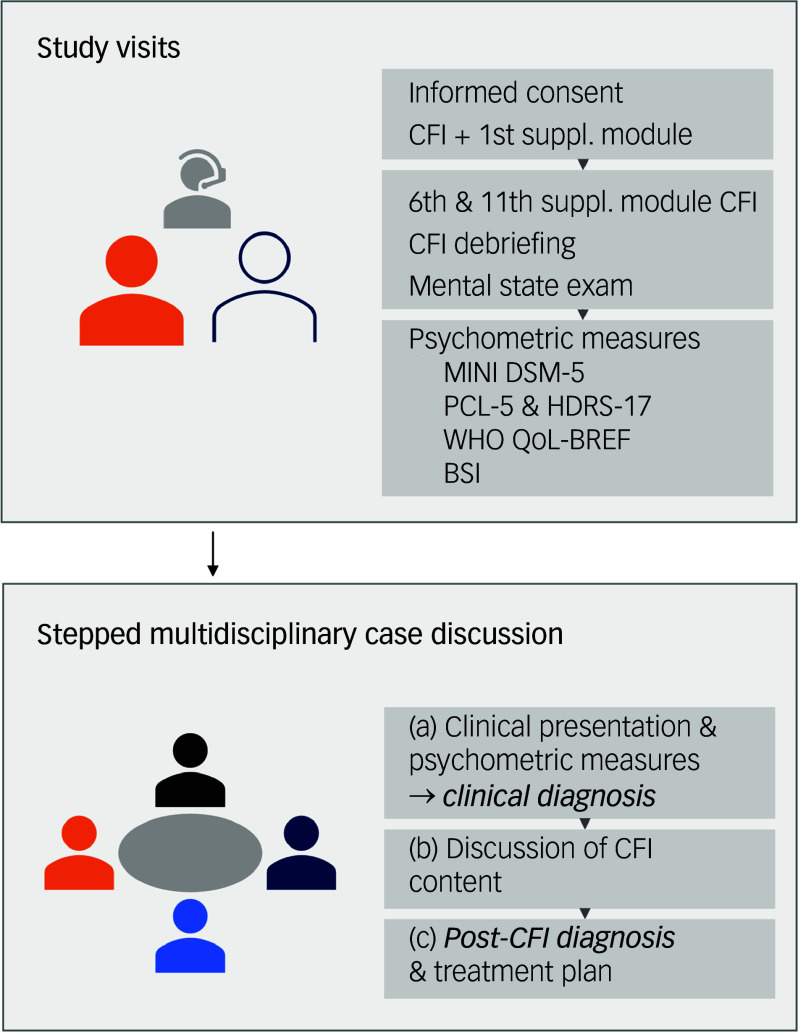
Overview of the study design, interviews and multidisciplinary case discussions. CFI, DSM-5 Cultural Formulation Interview; suppl., supplement; MINI DSM-5, Mini International Neuropsychiatric Interview for DSM-5; PCL-5, PTSD Checklist for DSM-5; HDRS-17, 17-item Hamilton Depression Rating Scale; WHO QoL-BREF, World Health Organization Quality of Life Questionnaire-BREF; BSI, Brief Symptom Inventory; adapted from: Claus et al.^26^

Following consent, the first author (L.C.) conducted a clinical assessment over three interviews of 1.5 h each (see Fig. 1). First, the researcher-psychiatrist administered the DSM-5 CFI and the first (explanatory) and sixth (cultural identity) supplementary modules. In the second session, the 11th supplementary module (migrants and refugees) was administered, followed by a debriefing and mental state examination. The final session included a structured psychiatric assessment using Mini-International Neuropsychiatric Interview (MINI) 7.0.2, the post-traumatic stress disorder (PTSD) Checklist for DSM-5 (PCL-5), the Hamilton Depression Rating Scale with 17 items (HDRS-17), the brief World Health Organization Quality of Life assessment (WHO QoL-BREF) and the Brief Symptom Inventory (BSI). These psychometric scales were used to mimic as fully as possible a standard clinical evaluation. The researcher could call upon interpreters’ support if needed.

This study applied a stepped methodological approach (see Fig. 1) to assess the CFI’s value in diagnostic assessment, based on previous CFI research experience with multidisciplinary case discussions.^8,27^ This approach includes structured case discussions with the interviewer (L.C.), the caregiver, a psychiatrist with expertise in cultural psychiatry (S.v.d.A.) and an independent psychiatrist. The interviewer maintained a neutral role, providing only information, while case discussions took place between the independent psychiatrist, the expert psychiatrist and the caregiver. The clinical diagnosis was initially based on the clinical information (main complaints, mental state examination, MINI diagnoses and symptom severity scores) (step 1). The CFI content was then discussed to determine the need and reasons for diagnostic revision (step 2), which led to a consensus final diagnosis (step 3). Clinical reports were written, presenting clinical and CFI data, followed by a structured summary of the case discussion notes on the clinical diagnostic process, cultural formulation and post-CFI diagnosis.

The authors assert that all procedures contributing to this work comply with the ethical standards of the relevant national and institutional committees on human experimentation, and with the Helsinki Declaration of 1975 as revised in 2013. All procedures were approved by the University of Antwerp’s ethics committee (no. BUN B3002022000005). Each participant received €15 per interview (maximum of three interviews). Data were managed using REDCap.^28^ NVivo release 1.7.1 (macOS; Lumivero, 2020; Denver, CO, USA; https://techcenter.qsrinternational.com/Content/nm20/standard_installation.htm) was used for the qualitative analysis, and SPSS version 29 (macOS; IBM Statistics, 2023; Natick, New York, NY, USA; https://www.ibm.com/support/pages/downloading-ibm-spss-statistics-29011) for quantitative analysis. Statistical significance was set at *P* = 0.05.

### Data analysis

#### Impact on diagnosis (quantitative analysis)

We defined diagnoses according to the DSM-5-TR diagnostic categories mentioned in the clinical and culturally sensitive evaluation during the ’stepped’ multidisciplinary case discussion. A participant might have one or more diagnoses at assessment time points. The prevalence of a shift between diagnostic categories was evaluated. The exact binomial and McNemar tests were used to determine whether the prevalence of diagnostic categories was significantly different between the clinical and culturally sensitive diagnoses. The impact of the CFI on the diagnostic process was then evaluated, showing change, narrowing or widening of diagnosis. ‘Diagnostic change’ was defined as an alteration in the diagnostic categorisation, which includes either a complete shift from one category to another or the removal of a category with the addition of a new one (misdiagnosis). ‘Diagnostic narrowing’ refers to reduction in the number of diagnostic categories without diagnostic change in the remaining categories (overdiagnosis). ‘Diagnostic widening’ implies the introduction of an additional diagnostic category without removal of any existing categories (underdiagnosis). Finally, post hoc comparisons of sociodemographic and clinical variables were conducted.

#### Value of the CFI (qualitative analysis)

The value of the CFI was evaluated through conventional content analysis of the multidisciplinary case discussion reports.^29^ Given the lack of prior knowledge, the analysis applied an inductive approach. This approach allowed for the generation of new insights and the development of a comprehensive understanding directly from the reports, without relying on preconceived categories. This study generally takes an epistemological social constructivist stance, because the knowledge generated is seen as being negotiated between people within the multidisciplinary case discussions.^30^ The analytical process followed the steps of inductive content analysis, as outlined by Elo et al.^31^ Following familiarisation, the first author (L.C.) assigned codes to meaning units then applied an open coding strategy focused on the manifest content. He consequently grouped and categorised the codes into sub- and major categories related to the added value of the CFI in the diagnostic process. The last author (S.v.d.A.) confirmed and validated the coding and categories. No new codes were added in the analysis of the final 15 reports. The large volume of coded reports ensures that the hypotheses are thoroughly stretched. To guarantee trustworthiness, categories were discussed with co-authors and any interpretation differences were resolved by consensus.

## Results

Following intake and consent, 67 participants agreed to participate, of whom 4 dropped out. Three minors felt there were too many questions, and one adult became suspicious of the personal nature of the CFI’s questions and withdrew. Data for one participant on WHO QoL-BREF, PCL-5 and BSI are missing due to the severity of his illness. Table 1 displays participants’ characteristics.

**Table 1:** Participant characteristics (*N* = 63)

	*n*	%
Gender		
Female	5	7.9
Male	58	92.1
Minor (15–18 years)	22	34.9
	Median	IQR
Age (years)	19	16–27
	*N* = 63	%
Interpreting assistance	58	92.1
Setting		
Hospital or therapeutic service	12	19.0
Reception centre	51	81.0
Country of origin		
Afghanistan	33	52.4
Palestine	6	9.5
Burundi	3	4.8
Iraq	3	4.8
Syria	2	3.2
Eritrea	2	3.2
Russia	2	3.2
Cameroon	1	1.6
Egypt	1	1.6
Gambia	1	1.6
Georgia	1	1.6
Ghana	1	1.6
Guinea	1	1.6
Macedonia	1	1.6
Morocco	1	1.6
Niger	1	1.6
Senegal	1	1.6
Somalia	1	1.6
MINI 7.0.2		
Psychotic disorder	3	4.9
Bipolar disorder	1	1.6
Depressive disorder	50	79.4
Anxiety disorder	26	41.3
OCD	1	1.6
PTSD	43	68.3
Substance use disorder	1	1.6
Personality disorder	1	1.6
No MINI diagnosis	6	9.5
	Mean	s.d.
Psychometric scales		
GSI (*n* = 62)	2.31	0.91
HDRS-17	16.70	6.94
PCL-5 (*n* = 62)	55.53	18.92
WHO QoL (*n* = 62)	38.71	25.78

MINI, MINI-International Neuropsychiatric Interview; OCD, obsessive–compulsive disorder; PTSD, post-traumatic stress disorder; GSI, Global Severity Index; HDRS-17, 17-item Hamilton Depression Rating Scale; PCL-5, PTSD Checklist for DSM 5; WHO QoL, World Health Organization Quality of Life Questionnaire; IQR, interquartile range.

A minority of participants (7.9%) identified as women. Most participants (*n* = 58, 92.1%) required the support of an interpreter. Around a third of the participants were minors (*n* = 22; 34.9%). The participants’ countries of origin are mainly situated in the Middle Eastern region: Afghanistan (52.4%), Palestine (9.5%), Iraq (4.8%) and Syria (3.2%). MINI interviews revealed a high prevalence of depressive (79.4%), anxiety (41.3%) and post-traumatic stress (68.3%) disorders. Symptom severity scales of mental illness (PCL-5, HDRS-17 and BSI) show high scores. The participants generally experienced their quality of life (WHO QoL-BREF) as below average.

### Impact of the CFI on diagnosis

**Fig. 2:**
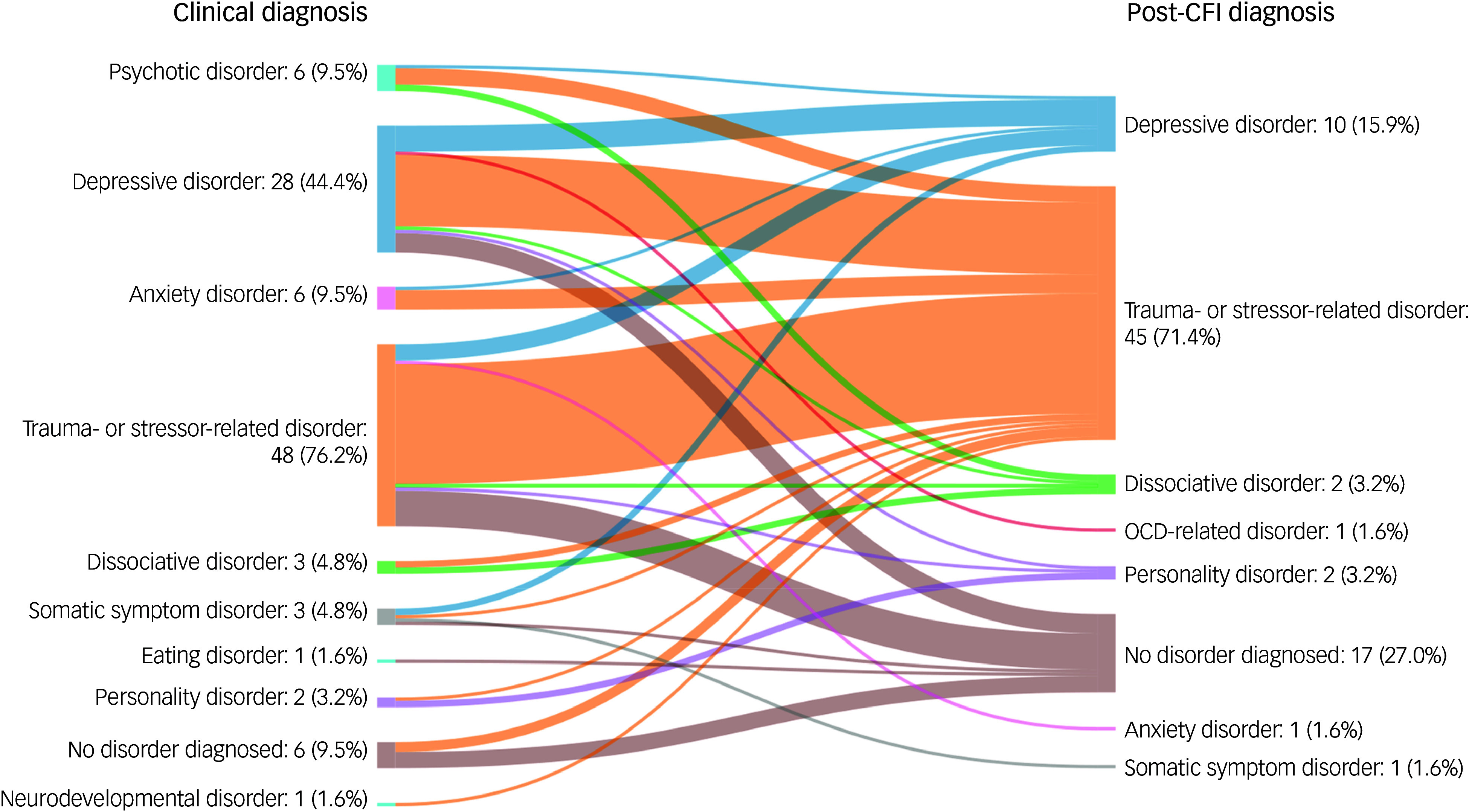
Sankey diagram of diagnostic shifts between clinical and CFI-informed diagnoses, expressed as total count (*n*) and percentage of cases (%); visual created with Sankey Matic. CFI, Cultural Formulation Interview; OCD, obsessive–compulsive disorder.

The Sankey diagram in Fig. 2 visually represents the diagnostic shifts between clinical and post-CFI diagnoses. Trauma- and stress-related disorders constituted the largest group in both clinical (*n* = 48, 76.2%) and post-CFI diagnoses (*n* = 45, 71.4%). In contrast, other common psychiatric disorders were diagnosed much less frequently following CFI assessment. Clinical evaluation identified six cases of psychotic disorders (9.5%) compared with none in the post-CFI assessment (*P* < 0.001). Depressive disorders were diagnosed in 28 cases (44.4%) clinically versus 10 cases (15.9%) post-CFI, with the proportion of depressive disorders in the CFI being approximately 36% (CI: 20.1–54.2%) of that found clinically (*P* < 0.001). Anxiety disorders were found in 6 cases (9.5%) clinically versus 1 case (1.6%) following CFI, also showing a lower post-CFI proportion (16.7%; CI: 1.9–55.8%, *P* = 0.125).

The number of cases with ‘no or other than DSM diagnosis’ shows an opposite trend because, clinically, 6 cases (9.5%) fell within this category whereas the CFI identified 17 cases (27.0%). The proportion of these cases was significantly lower among the clinical (35.3%; CI: 16.3–58.9%, *P* = 0.001) compared with post-CFI diagnoses. The group ‘no or other than DSM diagnosis’ includes several situations outside the bounds of DSM diagnoses. Clinical assessment revealed one case without the presence of psychopathology, while the CFI identified eight cases without psychopathology, which was determined by not meeting DSM criteria or absence of impact on daily functioning. Additionally, 4 clinical diagnoses involved grief, compared with 11 post-CFI, not meeting the criteria of persistent grief disorder (PGD). In the clinical diagnoses, one case remained undiagnosed due to a lack of clear diagnostic understanding. Four post-CFI cases were marked as ‘exceeding categorical DSM diagnoses’, because their suffering could not be adequately reflected by a categorical DSM diagnosis.

The patterns of diagnostic shift, as shown in Fig. 2, also reveal some key trends. Most participants with clinical psychotic or depressive disorders (83.3 and 78.6%, respectively) were assigned a trauma- or stressor-related disorder following CFI assessment. Notably, most trauma- or stressor-related diagnoses (81.3%) were confirmed following the CFI. Supplementary Table 2 offers more insights into the diagnostic shift per clinical diagnosis.

Analysis of the CFI’s impact on the diagnostic process revealed four distinct patterns: confirmation of diagnosis (36.5%), change of diagnostic category (27.0%), diagnostic narrowing (30.2%) and diagnostic widening (6.3%) (see Table 2). Post hoc analyses indicated no significant differences.

**Table 2:** Impact of the Cultural Formulation Interview on the diagnostic process

	Frequency	%	CI
Confirmation of diagnosis	22	34.9	24–47%
Change of diagnostic category	16	25.4	15–37%
Diagnostic narrowing	21	33.3	23–46%
Widening diagnosis	4	6.3	2–14%
Total	63	100.0	

### Value of the CFI in the diagnostic process

**Fig. 3:**
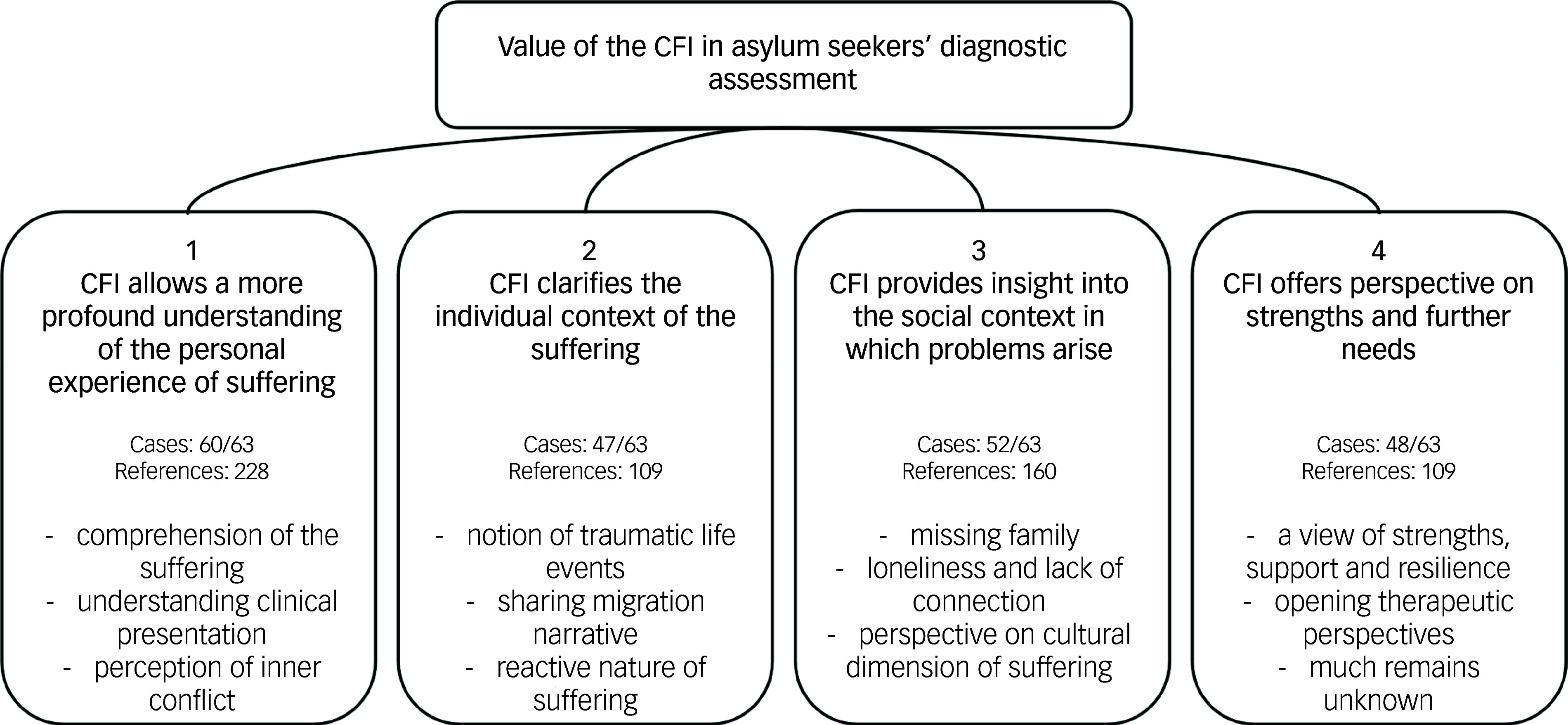
Themes and subthemes of the qualitative content analysis describing the added value of the Cultural Formulation Interview (CFI) in asylum seekers’ diagnostic assessment.

Qualitative content analysis distilled four key themes from the case discussion reports, illustrating the CFI’s added value in the diagnostic process. The themes and subthemes are outlined in Fig. 3 and described below. Text fragments from case discussion reports have been included to illustrate the findings.

The first theme indicates how the CFI allowed a more profound understanding of asylum seekers’ personal experience of suffering (see Table 3).

**Table 3:** Illustrative fragments for the first theme’s subthemes

Theme 1: the CFI allows a profound understanding of the personal experience of suffering	
Subtheme	Text fragments
Comprehension of the suffering	‘The CFI allows us to connect more with the young man’s suffering, (…), with the narrative being characterised by feelings of powerlessness and re-experiencing. We also gained more insight into the factors of stress, into the importance of own idioms of distress (possession) on his experience here and now.’ (P22, male, Afghanistan) ‘The CFI diagnosis exceeds categorical thinking in this participant, in whom hopelessness, lack of control and perspective are key in a context of numerous experiences of grief and loss.’ (P56, male, Afghanistan)
Understanding of the clinical presentation	‘Expressing the symptoms somatically also seems easier than having to find words to express her distress, for a patient who tends to relativise her own difficulties.’ (P53, female, Burundi) ‘Psychotic symptoms are perceived as rather secondary and part of decompensation. The emotional distance (…) can be seen as a ‘survival mode’ from the CFI narrative.’ (P62, male, Iraq) ’Such a threat to his integrity seems to have affected his confidence to such an extent that withdrawal and introversion can be seen as a logical consequence. (…) The normality of the reactions and absence of functional disturbance argues for a more contextual traumatic grief (…).’ (P16, male, Afghanistan)
Perception of inner conflict	‘[Name] experiences an internal conflict where he has difficulties adjusting expectations and obeying others, while at the same time struggling to resist or stand up for himself.’ (P40, male, Afghanistan) ‘It is striking how he holds on to what he considers good and valuable, e.g. taking care of his mother, struggling to fail, the value of his faith (…) while at the same time, the impression of an internal conflict arises because he cannot manage to meet his own ideals.’ (P49, male, Morocco) ‘The theme of being gay did not come up much during the treatment trajectory but was introduced as a very important identity theme during the CFI-interview (…), resulting in the image of someone who has never been able to exist.’ (P50, male, Ghana)

CFI, Cultural Formulation Interview.

First, the CFI facilitated comprehension of how participants had experienced their suffering, enabling them to better connect with asylum seekers’ personal understanding of their struggles. The CFI clarified the participants’ explanations of their illnesses and provided greater nuance. In many cases, the CFI created space for feelings of grief that were less addressed through the clinical descriptions. This helped identify the full width of psychic suffering that, in a few cases, exceeded a classic DSM-5 categorical diagnostic approach.

Second, the CFI enhanced the clinicians’ understanding of participants’ symptoms and clinical presentation. The CFI highlighted many participants’ difficulties in thinking about and expressing their problems. It enabled clinicians to evaluate the functional impact and disturbing effect of certain difficulties. The CFI framed observed behaviour within survival mechanisms and a quest for guidance. It also helped in understanding somatic symptoms as controlled expressions of psychological suffering. The CFI allowed reconsideration of clinically psychotic symptoms as expressions of an inability to cope with overwhelming distress, and of the transgression of the emotional repertoire rather than primary psychotic symptoms. It provided insights into personality functioning and emotion regulation mechanisms in several cases.

Third, the CFI allowed a change in the clinicians’ understanding of the underlying inner conflicts in many cases. It permitted them to notice disruptions in participants’ experience and development of identity; it helped them clarify what participants valued and to connect with their value-based attitudes. In some cases, the CFI helped clinicians explore participants’ desires, while in others it revealed the participants’ awareness of having no personal goals or desires. It permitted clinicians to notice participants’ experiences of discrimination, and of a lack of a right to exist, sometimes linked to the theme of sexual orientation.

The second theme focuses on the ability of the CFI to clarify the role of context in asylum seekers’ suffering (see Table 4). At first, the CFI allowed clinicians to become aware of participants’ traumatic life events, while also paying attention to the collective aspect of trauma. The CFI helped clinicians understand how participants connected their suffering to their traumatic experiences. In addition, migration experiences were also given more attention in CFI narratives, particularly through the 11th supplementary module for immigrants and refugees, than in traditional diagnostic settings. The CFI highlighted how events at various stages of migration had affected participants’ suffering.

**Table 4:** Illustrative fragments for the second theme’s subthemes

Theme 2: the CFI clarifies the role of trauma, migration and life events	
Subtheme	Text fragments
Notion of traumatic life events	‘The traumatic elements also appear more strongly in the formulation, (…) in particular the threat of the Taliban, (…) which he finds difficult to deal with.’ (P36, male, Afghanistan) ‘Based on the CFI (…) more insight into the collective aspect of the traumata.’ (P28, male, Palestine) ‘(…) and the burden of suffering due to the situations he experienced as life-threatening remains very high.’ (P25, male, Somalia)
Sharing migration narrative	‘The CFI migration module provide insight into the pre-, peri- and post-migratory difficulties he has faced and how each stage of migration has a significant impact on his experience of suffering today.’ (P35, male, Niger)
Reactive nature of suffering	‘Furthermore, the context of the (difficult life in the) reception centre also seems crucial to his suffering, with a great sense of helplessness in this situation. (…); based on the CFI information, [we] received a lot of information about how this context plays a central role in the manifestation of psychopathology.’ (P19) ‘[The CFI] draws an image of a boy who experiences a lot of misunderstanding (…). He is very preoccupied with his current situation, and this leads to suicidal thoughts. (…) The rich but burdened life history seems to have had a great impact on the participant.’ (P20)

CFI, Cultural Formulation Interview.

Last, the CFI highlighted how participants perceived their suffering as being reactive to current difficulties. The CFI enabled clinicians to better understand the realities faced by participants and the context in which their suffering emerged. The CFI also provided insights into how stressful life events impacted the current clinical situation, including the role of difficult living conditions in reception centres and the uncertain future within the asylum process. Additionall, the CFI helped clinicians recognise experiences of injustice and misunderstanding, by focusing on participants’ situations and contexts.

The third theme highlights the CFI’s value in providing insight into the social context in which asylum seekers’ problems arise (see Table 5). The CFI helped clinicians understand the significant impact of family separation on participants’ difficulties, in some cases perceiving the loss of relatives as a traumatic experience. The CFI additionally highlighted how feelings of responsibility towards family could contribute to increased stress and framed experiences of failure and inadequacy.

**Table 5:** Illustrative fragments for the third theme’s subthemes

Theme 3: the CFI provides insight into the social context in which asylum seekers’ problems arise	
Subtheme	Text fragments
Missing family	‘The difficulties seem to have started after his father’s murder (…) They also have the impression that the homesickness and missing of his mother and brothers is increasing. (…) From a great sense of duty to his family, he seems to want to build something positive, but he is constantly confronted with how external factors impede this.’ (P27, male, Afghanistan) ‘The traumatic loss of his grandfather, (…) are the main causes of the pain he feels (…) whereby he feels responsible for his parents and finds it more difficult to develop autonomy.’ (P26, male, Egypt)
Loneliness and lack of connection	‘By naming that at least in his homeland they had each other, we gain insight into a deep loneliness and a longing for connection to find support and meaning.’ (P38, male, Afghanistan) ‘Finally, the loss of all meaning, leaves little that gives him strength at present.’ (P41, male, Afghanistan) ‘He has long experienced feelings of insecurity, even before his birth (…) Due to the early separation from his parents, there seems to have been a halt in his emotional and psychological development.’ (P45, male, Afghanistan) ‘In the CFI narrative, he expresses an authentic great need for security and a parent figure who can offer him direction and support.’ (P37, male, Afghanistan)
Perspective on cultural dimension of suffering	‘The CFI highlights a form of cultural conflict between her love for Georgian culture and not being able to exist as a person in this culture.’ (P55, female, Georgia) ‘The CFI reveals how (participant) is proud of his origins and that reconnecting with his original culture is of great importance to him. This highlights the theme of acculturation, where he suffers from the distance to his country and culture of origin (…) where uprooting and acculturation seem to play an important role in the stress he is currently experiencing.’ (P59, male, Afghanistan) ‘Religion seems to be an important hold for him, as the only familiar thing left to him after leaving Afghanistan, while this also potentially implies vulnerability.’ (P37, male, Afghanistan)

CFI, Cultural Formulation Interview.

In many cases, the CFI facilitated engagement with participants’ feelings of loneliness and their lack of meaningful, safe connections. For some participants, the CFI revealed how the absence of meaning and connectedness had played a critical role in their distress. It also clarified the importance of proximity and emotional bonds for participants’ well-being. The CFI further provided clinicians with insight into how disrupted attachment and early experiences of insecurity had contributed to current difficulties. In some of the youngest participants, it was presumed that these early experiences had led to a halt in emotional and psychological development. The CFI also made more explicit to clinicians the participants’ need for a safe and secure environment.

In many cases, the CFI provided insights into the cultural dimensions of suffering, such as how forced migration had led to feelings of uprooting and grief over the loss of country and culture. In a few cases, the CFI opened space to explore how the acculturation process had affected participants’ difficulties. In a few cases, it facilitated the understanding of how aspects of cultural identity currently affect participants’ difficulties, such as identifying religion as an important support after losing their cultural framework.

The fourth theme maps out how the CFI offers a perspective on participants’ (lack of) strengths and further needs (see Table 6). The CFI enhanced clinicians’ understanding of asylum seekers’ strengths, coping strategies and resilience. It provided insights into various behaviours, such as avoiding discussing difficulties of the past, which was presumed to be a form of avoidant coping, or controlling, compulsive behaviours that were interpreted as an attempt to maintain control. The CFI uncovered a lack of coping skills and protective factors in some cases while highlighting a positive, future-focused mindset and active help-seeking behaviours in others.

**Table 6:** Illustrative fragments for the fourth theme’s subthemes

Theme 4: the CFI offers perspective on resilience and further needs	
Subtheme	Text fragments
A view of strengths, support and resilience	‘The CFI narrative offers a clear perspective on (…) his resilience, with some elements that he can contain himself (…) a young man looking for opportunities and solutions on his own.’ (P20, male, Afghanistan) ‘The importance of thinking positively, not dwelling on the past and looking to the future is a common thread throughout the CFI narrative. A positive and avoidant coping strategy seems to be present.’ (P1, male, Afghanistan) ‘Her compulsive and repetitive behaviours (e.g. cleaning her room) may serve to maintain some form of control in her life.’ (P30, female, Russia) ‘We also gain insight into how he lacks protective factors and coping skills to deal with the high level of stress he experiences, (…) which allows to understand how alcohol and drugs are implicated as dysfunctional coping.’ (P58, male, Iraq)
Open therapeutic perspectives	‘[the CFI assessment] allowed the participant to get in touch with his own authentic request for help.’ (P27, male, Afghanistan) ‘The CFI-narrative allowed (…) to better understand his needs.’ (P37, male, Afghanistan) ‘(…) he asks to develop a healthier coping strategy. (…) Focusing on what is important to him seems to open therapeutic perspectives.’ (P49, male, Morocco)
Much remains unknown	‘At the same time, much remains unknown, for example about early childhood trauma, relationships with support figures, and previous traumatic events. (…), much remains unclear.’ (P52, female, Macedonia) ‘Overall, however, the CFI’s contribution is limited for better understanding of the participant’s issues, (…) The question is raised how feasible it is to work with CFI with such seriously ill participants.’ (P34, male, Gambia)

CFI, Cultural Formulation Interview.

The CFI also helped open therapeutic perspectives, by facilitating the expression of an authentic request for help and the understanding of therapeutic needs. It clarified participants’ therapeutic goals and treatment options. However, the CFI could not provide a complete picture in some instances, leaving many questions unanswered and requiring further assessment. For a couple of severely ill participants, the feasibility of using the CFI was questioned.

## Discussion

This study aimed to evaluate the value of the CFI in the diagnostic assessment of asylum seekers. Our quantitative findings show a notable impact of the CFI on diagnostic outcomes. First, trauma- and stressor-related disorders were the most frequent diagnoses both before and after the CFI assessment. In contrast, diagnoses of psychosis and major depression significantly decreased (both *P* < 0.001) post-CFI. This aligns with previous findings of a strongly reduced prevalence of psychotic disorders following cultural assessment.^10^ In our study, the CFI recontextualised approximately 80% of symptoms initially interpreted as depression or psychosis within trauma- and stress-related disorders. This is supported by prior research showing that CFI-based discussions often lead to diagnostic shifts towards stress-related diagnoses.^27^ Second, the proportion of cases classified as ‘no or other than DSM diagnosis’ increased from 9.5 to 27.0% (*P* = 0.001). Prior research showed that cultural formulation, by contextualising distress, can help identify conditions as non-pathological.^8^ Finally, proportions of confirmation, change and narrowing of diagnoses were comparable, while diagnostic widening occurred less frequently. Also, in earlier research on cultural formulation with immigrants and refugees, diagnostic confirmation occurred in a minority of cases.^8^

The qualitative analysis complements the quantitative findings by highlighting four key themes that reflect the value of the CFI in the diagnostic process of asylum seekers. First, the CFI enhanced clinicians’ understanding of asylum seekers’ suffering, allowing for more nuanced illness narratives and bringing attention to often overlooked experiences such as grief. This aligns with previous research showing that cultural formulation improves understanding of individuals’ suffering and symptoms.^8,32,33^ The second theme highlighted the CFI’s role in revealing the impact of trauma, migration and life events on the diagnostic process. Rousseau et al (2020) confirmed that the CFI often uncovers significant traumas and stressors overlooked in initial assessments.^27^ In this context, our findings stressed the contribution of the supplementary modules, which were not subject to previous research. Our findings further emphasise the ability of the CFI to contextualise suffering within the realities faced by asylum seekers, while also framing difficulties as responses to abnormal situations.^34^ The third theme emphasised the role of the CFI in clarifying the social-relational context of asylum seekers’ difficulties. It highlighted the distress caused by family separation, loneliness, disrupted attachment and the impact of uprooting and acculturation. Our findings confirm the capacity of the CFI to understand social-relational suffering comprehensively.^20,27,34,35^ The fourth theme showed how the CFI, whose focus on strengths and resources was described earlier, provided valuable insights into asylum seekers’ needs, strengths and coping strategies.^27,32^ Despite its benefits, the CFI did not provide the clarity needed for a thorough diagnostic understanding in a few severely ill participants with already uncertain clinical diagnoses, for whom the narrative approach was less feasible. All themes reveal different aspects of asylum seekers’ suffering, which Cassel conceptualises as ‘the state of severe distress associated with events that threaten the intactness of a person’.^36^ Cassel’s aspects of suffering align with our first three themes. The first theme reflects his view of suffering as an experience of the whole person, reaching beyond a mind–body split, with the CFI revealing asylum seekers’ rich, nuanced and personal experiences. The second theme relates to threats to personal integrity, as shown by the CFI’s clarification of harmful contextual factors. The third theme echoes his idea that suffering may involve any aspect of the person, including social roles and relationships, with the CFI highlighting the social aspects of suffering.^36^

The mixed-methods approach allows a nuanced view of the CFI’s impact on asylum seekers’ diagnostic assessment. The shift toward stress-related disorders, and away from traditional DSM classifications, aligned with qualitative findings showing that the CFI offers a more nuanced and contextual understanding of mental health within asylum seekers’ life narratives and sociocultural contexts. Previous research highlighted how cultural formulation enabled significant revisions through contextualised illness narratives.^8^ Unlike our findings, a recent randomised controlled trial (RCT) noted a slight increase in depressive disorder diagnoses.^24^ Our qualitative findings clarified these differences, suggesting that the CFI may not lead to a universal pattern of diagnostic change, but instead promote diagnostic refinement through a more personalised and holistic understanding of suffering.

Our results showed how the CFI captured a broad range of experiences that fell outside traditional diagnostic criteria. This finding points to the concept of ‘category fallacy’, where DSM categories developed in specific cultural contexts may not fully apply to other groups.^37^ It criticises the construct validity of the DSM diagnoses, such as PTSD, which failed to fully cover the asylum seekers’ illness experience (e.g. grief) in our study. The limitations of these categorical frameworks, on which our quantitative analysis relied, must be considered. Alternative concepts such as cultural bereavement, traumatic grief and complex PTSD (cPTSD) may better reflect the suffering observed. Cultural bereavement could be recognised in themes such as loss of culture and acculturation difficulties, loss of meaning and uprooting. Traumatic grief, distinct from PTSD, was common in cases without clear DSM diagnoses. cPTSD was echoed through themes of early insecurity, difficulties in thinking about and expressing their problems, disrupted development and experiencing a lack of connection.

The findings from this study illustrate how the CFI fosters empathy by increasing clinicians’ understanding of individuals’ suffering, life history and circumstances. Previous research noted how the CFI encouraged curiosity and collaboration, emphasising its role in enhancing alliances and understanding.^32,38^ It described how the CFI strengthened clinician–patient relationships and prevented the essentialising of culture.^27^ Our results highlighted the individual nature of the subject’s story, helping clinicians avoid epistemic injustice. Epistemic injustice happens when people from marginalised groups are not seen as capable of producing knowledge and are left out of the process of ‘making sense’. In contrast, the CFI helps to ensure that people’s voices are heard and validated, and free from the dismissal caused by stereotypes or stigma.^39^ Cultural competence in mental healthcare reflects core ethical principles: respect, beneficence, non-maleficence and justice.^40^ Tools such as the CFI can support the effective implementation of these principles.

The causes of distress, as elicited by our findings, were deeply rooted in social contexts such as loss of social ties, as well as in persecution, displacement and the absence of basic care and protection. Earlier research highlighted the CFI’s role in promoting a complex vision of cultural and social understandings.^27^ It emphasises a certain mismatch with the biomedical model promoted by the DSM, which is criticised for ignoring social factors in mental illness and for its criteria reflecting social norms rather than being objective or value-free.^41,42^ This criticism resonates with Foucault’s critique on how psychiatry often enforces normative societal expectations under the guise of treatment, regulating behaviour rather than addressing the social roots of suffering. His analysis highlights the disciplinary nature of psychiatry, where power is exerted over the individual through societal institutions, including hospitals. This creates a dynamic where asylum seekers, with their unique sociopolitical struggles, are further marginalised by a system that fails to account for the structural causes of their suffering:

‘People’s misfortune must never be a silent remainder of politics. It grounds an absolute right to stand up and speak to those who hold power.’ (Michel Foucault)^43^

The CFI, by foregrounding personal narratives within their social context, counterbalances the depersonalising biomedical model. It underscores the need for a more socially oriented psychiatry that not only understands, but also advocates for, structural changes to address the root causes of suffering. This shift towards a more holistic, recovery-oriented approach is crucial in improving care for vulnerable populations such as asylum seekers.

### Limitations

To the best of our knowledge, this study is the first to investigate the CFI’s added value in asylum seekers’ psychiatric assessment, but it has certain limitations. A large portion of the participants were minors, raising questions about age-related differences in expressing distress and the validity of psychiatric diagnoses in young people. Although representative of the asylum seeker population in Belgium at the time of the study,^44^ the sample was predominantly male and included many Afghans. This might have impacted the transferability of the findings while still providing unique insights. Due to the referral for participation based on caregivers’ indication, a certain selection bias cannot be excluded. This study is among the first to publish on the clinical use of the supplementary modules. It was decided in advance to use the 1st (explanatory models), 6th (cultural identity) and 11th (immigrants and refugees) modules to achieve a comprehensive cultural and contextual evaluation. While migration was not clearly addressed in the clinical case descriptions, the 11th module thoroughly explored migration experiences and their effects on current difficulties. However, the study design did not allow for an in-depth exploration of the specific impact of these supplementary modules, making it harder to compare with earlier research. For this reason, the findings focus on cultural formulation as a method rather than as a specific questionnaire. This is one of the few CFI studies to mitigate language barriers with certified interpreters,^33^ possibly causing translation difficulties but also enhancing clinical representativeness. Earlier CFI studies showed a stronger effect in linguistically discordant encounters.^24^ Previous treatments, such as antipsychotics, may have affected symptom manifestation at the time of the interviews, and this was considered during the diagnostic assessment. Different psychometric instruments were used to strengthen the validity of clinical diagnoses. Substance use was reported as a coping mechanism but was never coded as a diagnosis because it did not fully meet DSM criteria. Additionally, a share of personality or developmental disorders may have remained undiagnosed due to a lack of more profound testing. Frequent uncertainty in clinical diagnoses, and sometimes post-CFI, highlighted the dynamic nature of diagnoses and the ongoing need for clarity, as described in the qualitative analysis. We addressed the potential presentation bias introduced by virtue of the main researcher (L.C.) being the interviewer and presenting clinician in the multidisciplinary case discussions, by presenting only the literal and structured summary of the interview transcripts. The main researcher (L.C.) did not interpret diagnoses. To ensure diverse perspectives and accuracy, each case was reviewed by at least three professionals: an independent psychiatrist, an expert psychiatrist (S.v.d.A.) and a caregiver. To control for information bias, the last author (S.v.d.A.) verified case reports and diagnostic codes.

The multi-method design, with minimal missing data or dropouts, enhanced the multifaceted understanding of the CFI’s added value through methodological triangulation. While the quantitative analysis provided thorough descriptive insights, the sample size restricted subgroup comparisons and pattern (e.g. cluster) analyses, necessitating larger studies. Qualitative content analysis allowed for broad exploration, while the research team’s prolonged engagement in asylum seekers’ mental healthcare ensured a profound understanding of the findings. Text fragments clarified the connection to the original data and enhanced the trustworthiness of the results. Methodological quality was further ensured through a pre-published protocol paper^26^ and by reporting according to the Consolidated Criteria for Reporting Qualitative Research guidelines.^45^ Our research team, clinical psychiatrists experienced in cultural psychiatry, is committed to advocating for the mental health of asylum seekers. The main researcher’s background as a White male resident psychiatrist and psychodynamic psychotherapist may have shaped interactions with participants, caregivers and analytic views. During the interviews, the main researcher focused on building relationships based on a sense of equality. This led to shared experiences of powerlessness, especially with unaccompanied minors, that urged us to recognise our limitations as researcher-caregivers within the current system and their situation. The project’s social constructivist approach recognised the possibility of personal bias.

### Clinical implications

This study underscored the CFI as a valuable tool in asylum seekers’ diagnostic assessment. Use of the CFI led to significant shifts in diagnostic categories and fostered the clinicians’ understanding of asylum seekers’ experiences. Its holistic perspective challenged traditional diagnostic frameworks and enhanced the understanding of individual needs and therapeutic goals, which can profoundly impact treatment strategies. This makes the findings of this study clinically very relevant, because incorrect diagnoses and treatments can severely compromise well-being and have lasting impacts. The CFI also provided a more profound understanding of the context in which psychopathology arises, often revealing that the primary issue is not the psychopathology itself. This recognition helps clinicians acknowledge their own sense of powerlessness in the asylum seekers’ situation, which can otherwise go unaddressed. Furthermore, case discussions offer an effective platform for openly acknowledging these experiences of powerlessness, helping to prevent the risk of over-medicalisation. Despite the strengths of the CFI, it sometimes fell short in providing a comprehensive understanding of the asylum seekers’ situation and diagnostic picture, particularly for severely ill individuals where the narrative approach was less feasible. These findings highlight the need to explore methods that ensure a culturally sensitive and thorough diagnostic assessment of asylum seekers with severe mental health illnesses. The migration module provided clinically relevant information on asylum seekers’ migration histories, supporting its value in diagnostic assessment and highlighting the need for further research on the use of supplementary modules. Further research should also address the lack of evidence on the CFI by exploring its use in specific populations, such as unaccompanied minors, and promoting greater involvement and participation in both research design and interpretation of findings. This study’s findings on using the CFI with asylum seekers shaped our understanding of their suffering and needs. From this perspective, these insights call for an evolution in the psychiatric diagnostic framework, moving beyond an exclusive focus on symptoms towards one that more fully incorporates the lived experiences and specific needs of asylum seekers.

This study’s findings may also direct the CFI’s future clinical implementation. First, it emphasises the need for CFI training to extend beyond CFI administration, focusing on sensitivity for context-specific suffering, trauma, migration and critical reflection on diagnostic categories. Second, integration into routine clinical assessment is essential, particularly for culturally diverse populations. Depending on subjects’ backgrounds, supplementary modules (e.g. for refugees) may add value. Limitations remain in regard to very young or severely ill individuals, warranting further exploration of culturally sensitive approaches in these more complex cases. Finally, the implementation of the CFI also calls for continued reflection on its ethical application and eventual adaptation

The CFI offers a promising approach to diagnostic challenges in cross-cultural settings and, although underutilised, shows significant potential in improving mental healthcare for asylum seekers. It prompts a conceptual reflection on how to understand psychopathology in asylum seekers. This study provides rare evidence of the CFI’s added value and highlights the need and possible strategies for further clinical implementation.

## Supporting information

Claus et al. supplementary material 1 Claus et al. supplementary material

Claus et al. supplementary material 2 Claus et al. supplementary material

## Data Availability

The data-set supporting the findings of this study is available from the corresponding author, L.C., upon reasonable request. The data are not publicly available for reasons of participants’ privacy.

## Supplementary material

The supplementary material is available online at https://doi.org/10.1192/bjo.2025.10828
