# Supplementary material for: A different lens on diagnosis: value of the CFI in asylum seekers’ psychiatric diagnostic assessment
Source: BJPsych Open. 2025 Sep 8;11(5):e202. doi: 10.1192/bjo.2025.10828 (PMC12451538; doi:10.1192/bjo.2025.10828)
Supplement: Claus et al. supplementary material 1 — Claus et al. supplementary material [file S2056472425108284sup001.docx]

| Participant ID | Gender | Country of birth | Interpreter | Age |
| --- | --- | --- | --- | --- |
| 1 | male | Afghanistan | Yes | 15 |
| 2 | male | Afghanistan | Yes | 15 |
| 3 | male | Afghanistan | Yes | 15 |
| 4 | male | Afghanistan | Yes | 16 |
| 5 | male | Afghanistan | Yes | 15 |
| 6 | male | Afghanistan | Yes | 15 |
| 7 | male | Afghanistan | Yes | 16 |
| 8 | male | Guinea | No | 23 |
| 9 | male | Afghanistan | Yes | 22 |
| 10 | male | Syria | Yes | 28 |
| 11 | male | Palestine | Yes | 29 |
| 12 | male | Eritrea | Yes | 23 |
| 13 | male | Afghanistan | Yes | 27 |
| 14 | female | Burundi | Yes | 27 |
| 15 | male | Afghanistan | Yes | 17 |
| 16 | male | Afghanistan | Yes | 17 |
| 17 | male | Afghanistan | Yes | 27 |
| 18 | male | Afghanistan | Yes | 18 |
| 19 | male | Afghanistan | Yes | 16 |
| 20 | male | Afghanistan | Yes | 16 |
| 21 | male | Afghanistan | Yes | 15 |
| 22 | male | Afghanistan | Yes | 15 |
| 23 | male | Afghanistan | Yes | 24 |
| 24 | male | Egypt | Yes | 21 |
| 25 | male | Somalia | Yes | 23 |
| 26 | male | Cameroon | No | 20 |
| 27 | male | Afghanistan | Yes | 22 |
| 28 | male | Palestine | Yes | 19 |
| 29 | male | Afghanistan | Yes | 17 |
| 30 | female | Russia | Yes | 30 |
| 31 | male | Afghanistan | Yes | 15 |
| 32 | male | Afghanistan | Yes | 27 |
| 33 | male | Syria | Yes | 16 |
| 34 | male | Gambia | Yes | 30 |
| 35 | male | Niger | Yes | 19 |
| 36 | male | Afghanistan | Yes | 26 |
| 37 | male | Afghanistan | Yes | 20 |
| 38 | male | Afghanistan | Yes | 18 |
| 39 | male | Palestine | Yes | 27 |
| 40 | male | Afghanistan | Yes | 17 |
| 41 | male | Afghanistan | Yes | 18 |
| 42 | male | Burundi | No | 26 |
| 43 | male | Senegal | Yes | 18 |
| 44 | male | Afghanistan | Yes | 15 |
| 45 | male | Iran | Yes | 15 |
| 46 | male | Afghanistan | Yes | 15 |
| 47 | male | Palestine | Yes | 18 |
| 48 | male | Afghanistan | Yes | 19 |
| 49 | male | Morocco | Yes | 18 |
| 50 | male | Ghana | No | 28 |
| 51 | male | Palestine | Yes | 29 |
| 52 | female | Macedonia | Yes | 30 |
| 53 | female | Burundi | Yes | 22 |
| 54 | male | Palestine | Yes | 27 |
| 55 | female | Georgia | Yes | 26 |
| 56 | male | Afghanistan | Yes | 21 |
| 57 | male | Russia | Yes | 29 |
| 58 | male | Iraq | Yes | 21 |
| 59 | male | Afghanistan | Yes | 15 |
| 60 | male | Afghanistan | Yes | 15 |
| 61 | male | Iraq | No | 30 |
| 62 | male | Iraq | Yes | 19 |
| 63 | male | Eritrea | Yes | 27 |

Supplementary Table 1: participant characteristics: gender (male/female), country of origin, use of an interpreter (yes/no), age (years)
