# Supplementary material for: A different lens on diagnosis: value of the CFI in asylum seekers’ psychiatric diagnostic assessment
Source: BJPsych Open. 2025 Sep 8;11(5):e202. doi: 10.1192/bjo.2025.10828 (PMC12451538; doi:10.1192/bjo.2025.10828)
Supplement: Claus et al. supplementary material 2 — Claus et al. supplementary material [file S2056472425108284sup002.docx]

| Clinical Diagnosis | N | CFI Diagnosis | N | % |
| --- | --- | --- | --- | --- |
| Psychotic disorder | 6 |  |  |  |
|  |  | Depressive disorder | 1 | 16.7 |
|  |  | Trauma or stressor-related disorder | 5 | 83.3 |
|  |  | Dissociative disorder | 2 | 33.3 |
| Depressive disorder | 28 |  |  |  |
|  |  | Depressive disorder | 8 | 28.6 |
|  |  | OCD related disorder | 1 | 3.6 |
|  |  | Trauma or stressor-related disorder | 22 | 78.6 |
|  |  | Dissociative disorder | 1 | 3.6 |
|  |  | Personality disorder | 1 | 3.6 |
|  |  | No or other than DSM diagnosis | 6 | 21.4 |
| Anxiety disorder | 6 |  |  |  |
|  |  | Depressive disorder | 1 | 16.7 |
|  |  | Trauma or stressor-related disorder | 6 | 100 |
| Trauma or stressor-related disorder | 48 |  |  |  |
|  |  | Depressive disorder | 5 | 10.9 |
|  |  | Anxiety disorder | 1 | 2.2 |
|  |  | Trauma or stressor-related disorder | 39 | 81.3 |
|  |  | Dissociative disorder | 1 | 2.2 |
|  |  | Personality disorder | 1 | 2.2 |
|  |  | No or other than DSM diagnosis | 12 | 25.0 |
| Dissociative disorder | 3 |  |  |  |
|  |  | Trauma or stressor-related disorder | 2 | 66.6 |
|  |  | Dissociative disorder | 2 | 66.6 |
| Somatic symptom disorder | 3 |  |  |  |
|  |  | Depressive disorder | 2 | 66.6 |
|  |  | Trauma or stressor-related disorder | 1 | 33.3 |
|  |  | Somatic symptom disorder | 1 | 33.3 |
|  |  | No or other than DSM diagnosis | 1 | 33.3 |
| Eating disorder | 1 |  |  |  |
|  |  | No or other than DSM diagnosis | 1 | 100 |
| Personality disorder | 2 |  |  |  |
|  |  | Trauma or stressor-related disorder | 1 | 50.0 |
|  |  | Personality disorder | 2 | 100 |
| No or other than DSM diagnosis | 6 |  |  |  |
|  |  | Trauma or stressor-related disorder | 3 | 50.0 |
|  |  | No or other than DSM diagnosis | 5 | 83.3 |

Supplementary table 2: diagnostic shift per clinical diagnosis
